# Trunk surface agarwood-inducing technique with *Rigidoporus vinctus*: An efficient novel method for agarwood production

**DOI:** 10.1371/journal.pone.0198111

**Published:** 2018-06-01

**Authors:** Xuyu Chen, Yangyang Liu, Yun Yang, Jian Feng, Peiwei Liu, Chun Sui, Jianhe Wei

**Affiliations:** 1 Key Laboratory of Bioactive Substances and Resources Utilization of Chinese Herbal Medicine, Ministry of Education & National Engineering Laboratory for Breeding of Endangered Medicinal Materials, Institute of Medicinal Plant Development, Chinese Academy of Medical Sciences & Peking Union Medical College, Beijing, China; 2 Hainan Provincial Key Laboratory of Resources Conservation and Development of Southern Medicine, Hainan Branch of Institute of Medicinal Plant Development, Chinese Academy of Medicinal Sciences & Peking Union Medical College, Haikou, Hainan, China; 3 Key Laboratory of State Administration of Traditional Chinese Medicine for Agarwood Sustainable Utilization, Hainan Branch of Institute of Medicinal Plant Development, Chinese Academy of Medicinal Sciences & Peking Union Medical College, Haikou, Hainan, China; Tallinn University of Technology, ESTONIA

## Abstract

Only when *Aquilaria* spp. or *Gyrinops* spp. trees are wounded, due to insect attack, or microbial invasion, agarwood can be successfully induced. In the present study, a fungus which can induce agarwood formation efficiently was isolated and a suitable method for its application to induce agarwood formation was developed. *Rigidoporus vinctus* was isolated from the inner layers from infectious *A*. *sinensis* trees. When the fermentation liquid of fungi inoculated back to *A*. *sinensis* tree, agarwood was found to be induced. In addition, a novel method called trunk surface agarwood-inducing technique (Agar-Sit) was developed to produce agarwood with *R*. *vinctus*. The alcohol soluble extract content of the agarwood, up to 38.9%, far higher than the requirement (10%) in Chinese Pharmacopoeia and the six characteristic compounds of agarwood used as Chinese Medicinal Materials were all detected. Their relative percentages of the sesquiterpenes in the essential oil were 22.76%. This is the first report of the Agar-Sit and also the application of *R*. *vinctus* in agarwood induction. According to the results, when the combination of Agar-Sit and *R*. *vinctus* is used agarwood can be induced with high yield and good quality.

## Introduction

Agarwood, the resinous wood of *Aquilaria* spp. or *Gyrinops* spp. trees [1–3], is highly valued for its extensive use in medicine, perfumes, and incense across Asia, Middle East and Europe [4]. Agarwood is one of the famous traditional medicine for sedative, carminative, and anti-emetic effects in China, produced from *Aquilaria sinensis*. The wild resources of *Aquilaria* spp. *and Gyrinops* spp. are coming to be endangered and has been placed in the Appendix II list of the Convention on International Trade in Endangered Species of Wild Fauna and Flora since 2004 [5–6]. The natural formation of agarwood with high quality needs a few years or even decades after the tree is damaged by certain external factors, such as lightning strike, animal grazing, insect attack, or microbial invasion [7]. Along with the continuously decreasing supply of wild agarwood and in order to meet market demand, much effort has been devoted to agarwood production. In 2010, an effective technique called the whole-tree agarwood-inducing technique (Agar-Wit) was developed in our laboratory [8]. When Agar-Wit is used, Agarwood formation is mainly inside the trunk, but the surface of the trunk wood is not fully utilized. Of note, fungi have been found to play a certain role in promoting agarwood formation. Back in 1935, Burkill recognized that fungal infections to trees were the cause of agarwood formation [9]. In 1952, Bhattacharya et al. reaffirmed there was a relationship between agarwood formation and fungal infections [10]. In 1976, the researchers from Guangdong Institute of Botany reported for the first time that fungi infection back to *A*. *sinensis* led to the agarwood formation [11]. In 1998, Qi et al. reported that *Menanotus flavolives* can accelerate agarwood formation in *A*. *sinensis* [12]. In 2005, Subeham et al. inoculated *Fusarium laseritum* into the holes on the trunk of *Aquilaria* spp. trees and obtained agarwood one year later [13]. In 2011, Xu reported that *Fusarium* sp. promoted agarwood formation one year after fungi inoculation [14]. In 2017, the fungi of *Lasiodiplodia theobromae* which can promote agarwood formation was reported by the research [15].

Fungi above mentioned can induce agarwood formation, but a suitable process to inject the fungi into the trunk or branches of an *Aquilaria* tree to induce agarwood formation with high efficiency is to be identified. The most popular method that has been used is to punch holes into the trunk and then to subsequently inject fungi into the trunk. For example, Huang et al. punched holes of 0.4–0.9 cm in diameter and 4 cm in depth, and agarwood formed six months after inoculation of *Schizophyllum commune* suspension into these holes [16]. Guo et al. punched holes of 1 cm in diameter and 3 cm in depth into the trunk, inoculated the strains of *Microsphaeropsis* sp., *Xylaria* sp., and *Lasiodiplodia* sp. into the holes, and observed agarwood formation two months later [17] Blanchette et al. drilled holes in the tree trunk and kept the wound open by putting a small piece of plastic pipe in the hole, and then inoculated natural fungus growing with CA-Kits [18].

Until now, all the fungi being tested for promoting agarwood formation have not been used widely in the agarwood production. The underlined causes may partially lie in the low efficiency and hard application, as well as the poor yield and quality of agarwood, thus produced. Here, the study reports a fungus with strong infection ability that accelerates the agarwood formation. Using the fungus, an efficient agarwood production method called Trunk Surface Agarwood-Inducing Technique (Agar-Sit) was also invented which realized agarwood formation on the trunk surface. This technique avoids severe damage to trees, and also allows for easy agarwood collection. Meanwhile, a considerably high yield and good quality of agarwood were available, along with the feasibility of repeated agarwood formation from the same tree.

## Materials and methods

### Plant materials for fungi isolation

**Fig 1:**
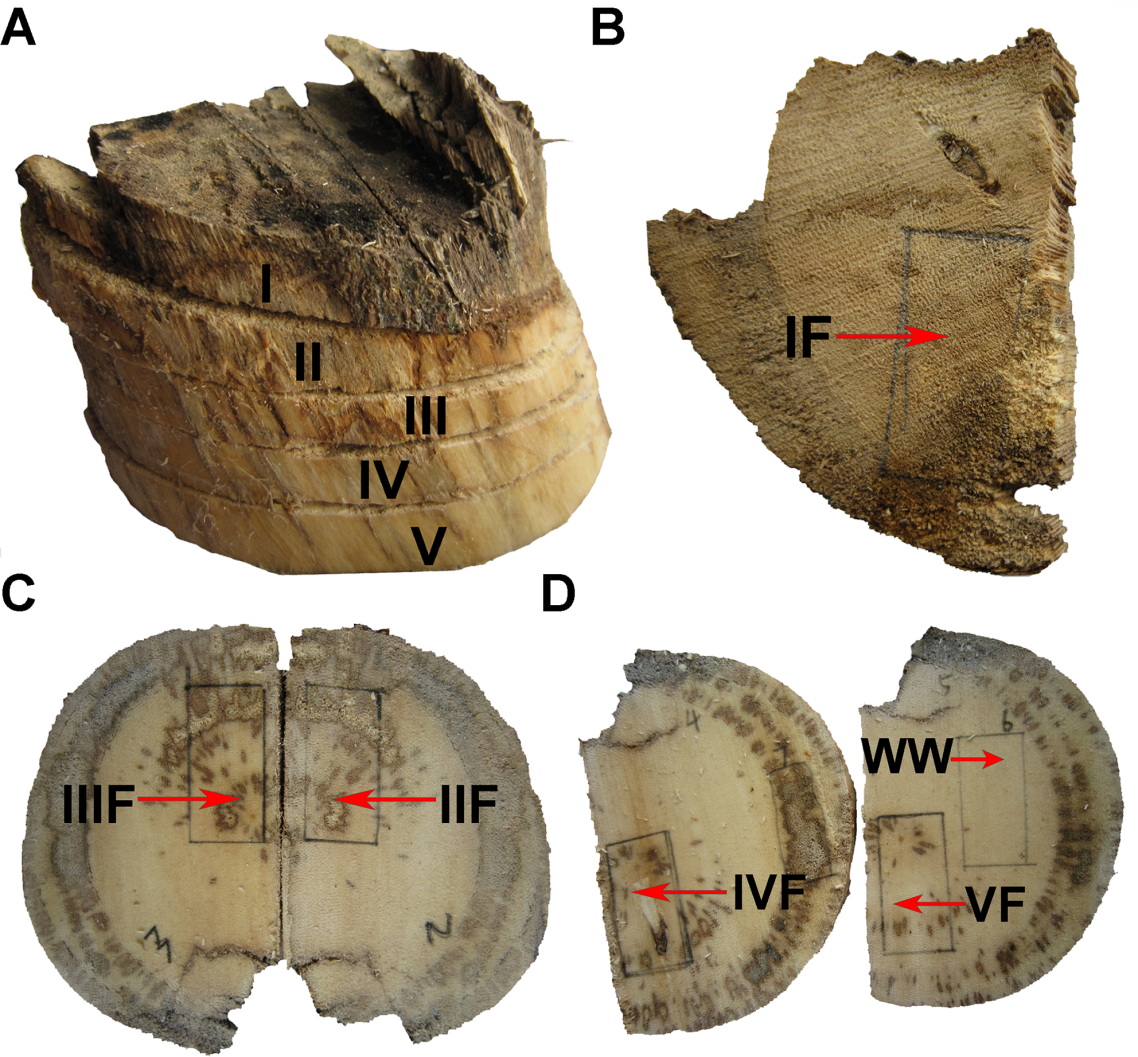
The wood material used for fungal isolation. A: Five layers (I, II, III, IV, V) were sliced. B, C, and D: Five rectangular chunks (IF, IIF, IIIF, IVF, VF) and a control (WW) were cut from the aforementioned five layers.

A 5 cm-diameter branch sprouting from the base of the trunk was cut off from an eight-year-old *A*. *sinensis* tree for agarwood production in Yanfeng town, Hainan Provinces of China (N19°57.843′, E110°34.134′ and 37 m above sea level). Three years later, a thick agarwood layer formed beneath the wounded interface. In order to harvest agarwood and isolate potential agarwood-promoting fungi, the agarwood-bearing branch was cut down. Five layers were spliced downstream the original cutting site, 1 cm thick each. For each layer, a rectangular chunk of 1.5 cm ×1 cm was used for fungal isolation. Another chunk of white wood from the fifth layer was used as control (Fig 1).

### Isolation of fungi

The chunks of 1.5×1×1 cm were first washed thoroughly in running tap water, then immersed in 70% ethanol for 3–5 min and 10% sodium hypochlorite for 5 min, and finally rinsed in sterile distilled water for three times. The sterilized samples were cut into small pieces using a cork borer, which were placed in 90 mm-diameter Petri dishes that contained potato dextrose agar (PDA) medium (potato 200 g, glucose 20 g and agar 15 g in one liter) with 100 mg ampicillin l-1 to suppress bacterium contamination. Next, all the Petri dishes were placed in a light chamber at 12 h light/dark cycles at 28°C ± 2°C. Five days later, new fungal colonies were monitored or picked out onto new PDA media every day, and this process lasted at least 2 weeks until all the fungi were separated as single colonies. Individual fungal colonies were picked from the edge with a sterile fine tipped needle and transferred onto PDA media. After subculture, these fungi were stored at Hainan Branch Institute of Medicinal Plant Development, Chinese Academy of Medical Sciences. The isolate of *R*. *vinctus* ASAF02 (CGMCC No. 9594) was deposited as living cultures in the China General Microbiological Culture Collection Center. The fungi diversity in different layers were evaluated by Shannon’s diversity index and Shimpson’s diversity index(1/D) index using R version 3.4.1 software.

### Method for cultivation and identification of fungi

The spore was observed with a scanning electron microscope (OLYMPUS SZX16) at Hainan Academy of Agricultural Sciences. For those fungi that did not sporulate, pore production by light culture had to be induced for about one month. The fresh Mycelia were inoculated into a 100 mL Erlenmeyer flask containing 50 mL liquid potato dextrose (PD) medium and cultured in a shaking incubator at 150 rpm/min for 7 days in darkness at 28°C. The fresh Mycelia of each fungus were used for DNA extraction with a plant genomic DNA kit (TIANGEN, CHINA). Primers ITS1 (5′-TCCGATGGTGAACCTGCGG-3′) and ITS4 (5′-TCCTCCGCTTATTGATATGC-3′) were used to clone the ITS sequences of each fungus. The amplification was performed in a 25 μl reaction volume containing 100 ng template DNA, 1 μl of 10 pmol of each primer, and 12.5 μl of 2 × PCR Master Mix (TIANGEN, China). The thermal cycling program was referred to the report of Chen et al. [19]. A negative control using water instead of template DNA was included in the amplification process. PCR products with distinct bands were sequenced using the primer pairs ITS1 and ITS4 on an ABI 3730 XL sequencer (SANGON, China). The representative isolates were listed in Table 1.

**Table 1:** The isolates identified from different layers.

Layers	Identified fungi	Representative isolates Genbank accession No.	Number of isolates	Shannon’s diversity index	Shimpson’s diversity index (1/D)
**I**	*Pleosporales* sp.	MF579569	40	1.5	0.67
	*Alternaria* sp.	MF579570	1		
	*Endomelanconiopsis* sp.	MF579571	4		
	*Fusarium* sp.	MF579572	18		
	*Trichothecium* sp.	MF579573	2		
	*Daldinia eschscholtzii*	MF579574	7		
	*Xylaria* sp.	MF579575	1		
	*Pestalotiopsis* sp.	MF579576	1		
	*Phomopsis* sp.	MF579577	2		
	*Nigrospora oryzae*	MF579578	2		
**II**	*Pleosporales* sp.	MF579579	18	1.06	0.64
	*Fusarium* sp.	MF579580	23		
	*Rigidoporus vinctus*	MF579581	11		
**III**	*Pleosporales* sp.	MF579582	35	0.70	0.41
	*Fusarium* sp.	MF579583	2		
	*Rigidoporus vinctus*	MF579584	11		
**IV**	*Pleosporales* sp.	MF579585	19	1.11	0.64
	*Phomopsis* sp.	MF579586	8		
	*Diaporthe* sp.	MF579587	1		
	*Rigidoporus vinctus*	MF579588	20		
**V**	*Pleosporales* sp.	MF579589	21	0.99	0.58
	*Alternaria* sp.	MF579590	1		
	*Fusarium* sp.	MF579591	3		
	*Rigidoporus vinctus*	MF579592	16		
**WW**	*Pleosporales* sp.	MF579593	30	0.14	0.06
	*Coprinopsis* sp.	MF579594	1		

### The ability test of agarwood induction of isolated fungi

*R*. *vinctus* was most frequently isolated from the *A*. *sinensis*, *R*. *vinctus* was cultured in the liquid PDA medium for seven days. The mycelium was filtered, and the remaining fermentation liquid was injected into the wood of three-year-old *A*. *sinensis* trees with Agar-Wit technique. Three trees were used for each treatment. Two months later, the woods were cut at 10 cm above and below the injected sites (at the trunk, 30 cm above the ground). Agarwood-like materials were separately collected.

### TLC (Thin Layer Chromatography) and alcohol soluble extracts content assay of agarwood

The TLC was conducted as previously reported [15]. Chromone isolated previously in the laboratory was used as standard constituents (ST). Alcohol soluble extracts content was assayed according to the procedure given in Chinese Pharmacopoeia [20]. The percentage of alcohol soluble extracts was calculated with reference to the dried powder. The assay was repeated twice.

### HPLC (High-performance liquid chromatography) method for detection of the characteristic components of agarwood

The standard agarwood sample was bought from the National Institutes for Food and Drug Control in China. All the samples preparation was according to ref. [21] with slight modification. An aliquot of 0.2 g of the sample (60 meshes) was extracted with 10 mL of 95% ethanol aqueous in an ultrasonic water bath for 60 min. The solution was cooled to room temperature and adjusted to the original weight with 95% ethanol aqueous. The obtained solution was filtered through a membrane filter (0.45 μm) prior to injection to the HPLC system. The chromatographic condition was according to ref. [22] with slight modification. A Diamonsil C18 column (4.6 mm×250 mm, 5 μm) was employed for the separation at 30°C. A gradient elution of A (acetonitrile) and B (0.1% aqueous formic acid) was used as follows: 0–10 min, 15%-20% A; 10–19 min, 20%-23% A; 19-21min, 23%-33% A; 21–39 min, 33% A; 39–40 min, 33%-35% A; 40–50 min, 35% A; 50–50.1 min, 35%-95% A; 50.1–55 min, 95% A. The flow rate was 0.7 mL/min and the detection was set at 252 nm.

### HPLC method for quantification of agarotetrol of agarwood

Agarotetrol standard, bought from the National Institutes for Food and Drug Control in China, was dissolved in ethanol as 60 μg/mL. The sample preparation and HPLC condition were similar to the above method described, except that the following gradient elution of A (acetonitrile) and B (0.1% aqueous formic acid) was used: 0–10 min, 15%-20% A; 10–19 min, 20%-23% A; 19-21min, 23%-33% A; 21–25 min, 33% A; 25.1–35 min, 95% A.

### GC-MS (Gas Chromatograph-Mass Spectrum) method for essential oil in the agarwood

The essential oil was extracted according to ref. [22] with slight modification. The dried powder of agarwood (5 g) was soaked in distilled water and then refluxed for 6 h. The essential oil (10 μL) was dissolved in n-hexane, filtered and stored in sealed containers under refrigeration prior to analysis. The GC–MS was run with an Agilent Technologies 7890A-5775C (USA). An HP-5MS capillary column (30 m × 0.25 mm×0.25 μm) was used. The injector temperature was 240°C, and the injection was performed in a splitless mode. High-purity (over purity 99.99%) nitrogen was used as the carrier gas. The mass conditions were set as follows: Ionization mode with EI, the ionization energy of 70 eV, ion source temperature of 230°C, 4 bar temperature at 150°C, and the scan range between 50.0 and 300 m/z. Compound identification was done by comparing the NIST library data of the peaks with those reported in the literature, and mass spectra of the peaks with literature data. Percentage composition was computed from the GC peak areas with the DB-5 MS column without applying correction factors.

## Results

### Fungi distribution in different wood layers

In the wild field, a phenomenon was found in which the suspected fungi infected the wood to induce agarwood formation in *A*. *sinensis*. Through microscopic observation, the fungal hyphae had been found in the wood sample. A total of 298 fungi were isolated from different layers of the wood samples. From the first layer, 78 isolates were identified, including 40 *Pleosporales* spp., 18 *Fusarium* spp., and 20 other species. From the second layer, 52 isolates were identified, including 18 *Pleosporales* spp., 23 *Fusarium* spp., and 11 *R*. *vinctus*. From the third layer, 48 isolates were identified, including 35 *Pleosporales* spp., 2 *Fusarium* spp., and 11 *R*. *vinctus*. From the fourth layer, 48 isolates were identified, including 19 *Pleosporales* spp., 20 *R*. *vinctus*, 8 *Phomopsis* spp., and 1 *Diaporthe* sp. From the fifth layer, 41 isolates were identified, including 21 *Pleosporales* spp., 16 *R*. *vinctus*, 3 *Fusarium* spp., and 1 *Alternaria* sp. The white wood in the fifth layer was taken as control (WW), where 30 *Pleosporales* spp. and 1 *Coprinopsis* sp. were identified (Table 1). A highest and lowest fungi diversity were represented in the outer first layer and healthy wood layer respectively which were shown by statistical analysis with Shannon’s diversity index and Shimpson’s diversity index(1/D) index. This is well accordance with the real situation.

**Fig 2:**
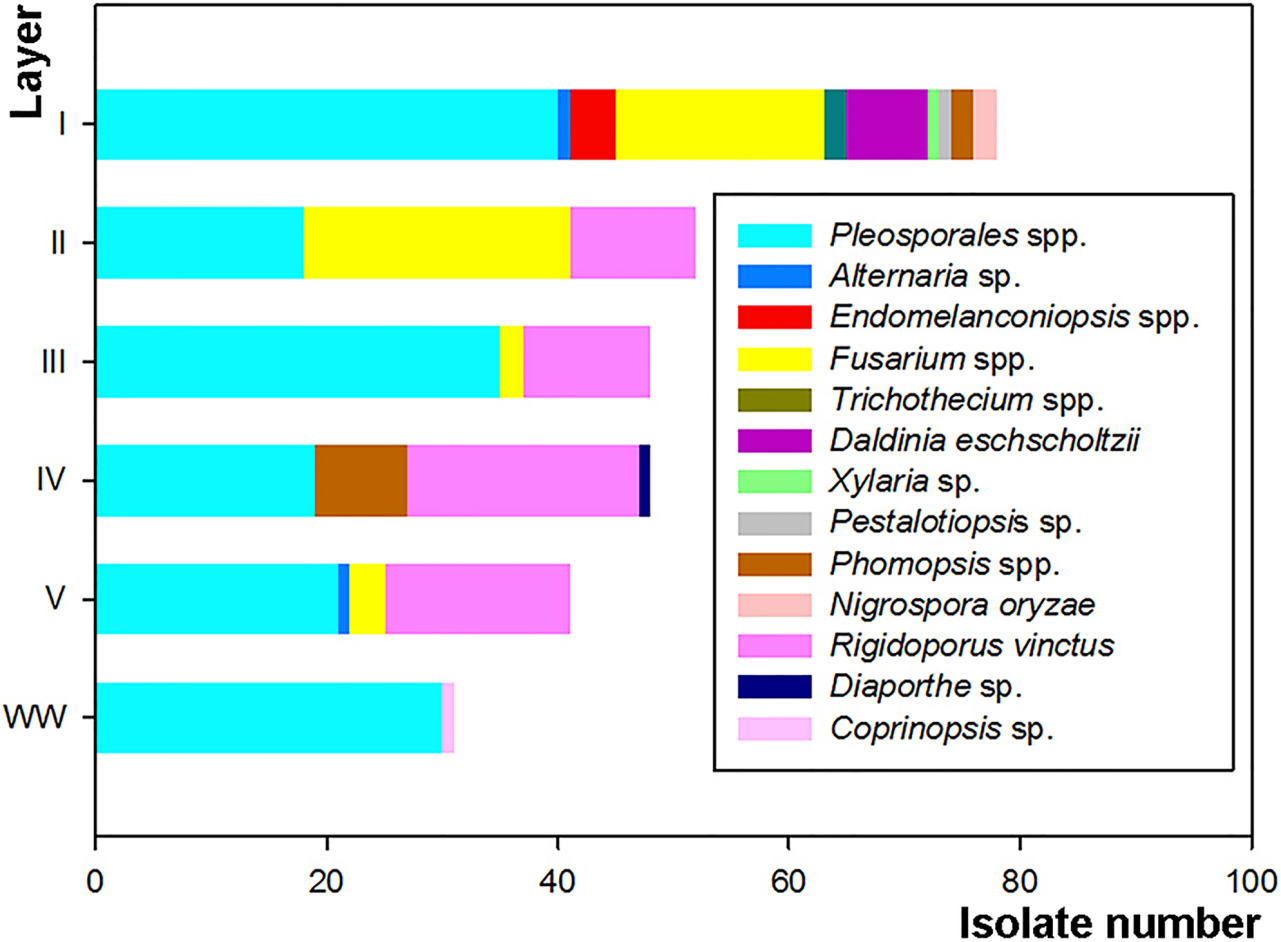
The fungal distribution in different layers of wood beneath the cutting site.

The fungal distribution showed that a large amount of *Fusarium* spp. existed in most layers except in the fourth and WW layers. In addition, the amount of *Fusarium* spp. in the third and fifth layers was much smaller than in the first and second layers. A large number of *Pleosporales* spp. were found in all layers including WW control layer, so it was observed that *Pleosporales* spp. might be the endophytic fungi and therefore cannot promote agarwood formation. The isolates of *R*. *vinctus* were found in the first, second, third, and fourth layer as well as beneath the fifth layer, except for the WW control layer. Of note, the amount of *R*. *vinctus* in the third and fourth layers was larger than in the first and second layers (Fig 2). Considering the distribution of the agarwood-like material in all the layers, it was assumed that *R*. *vinctus* might play a certain role in promoting agarwood formation.

### *R*. *vinctus* in promoting agarwood formation

**Fig 3:**
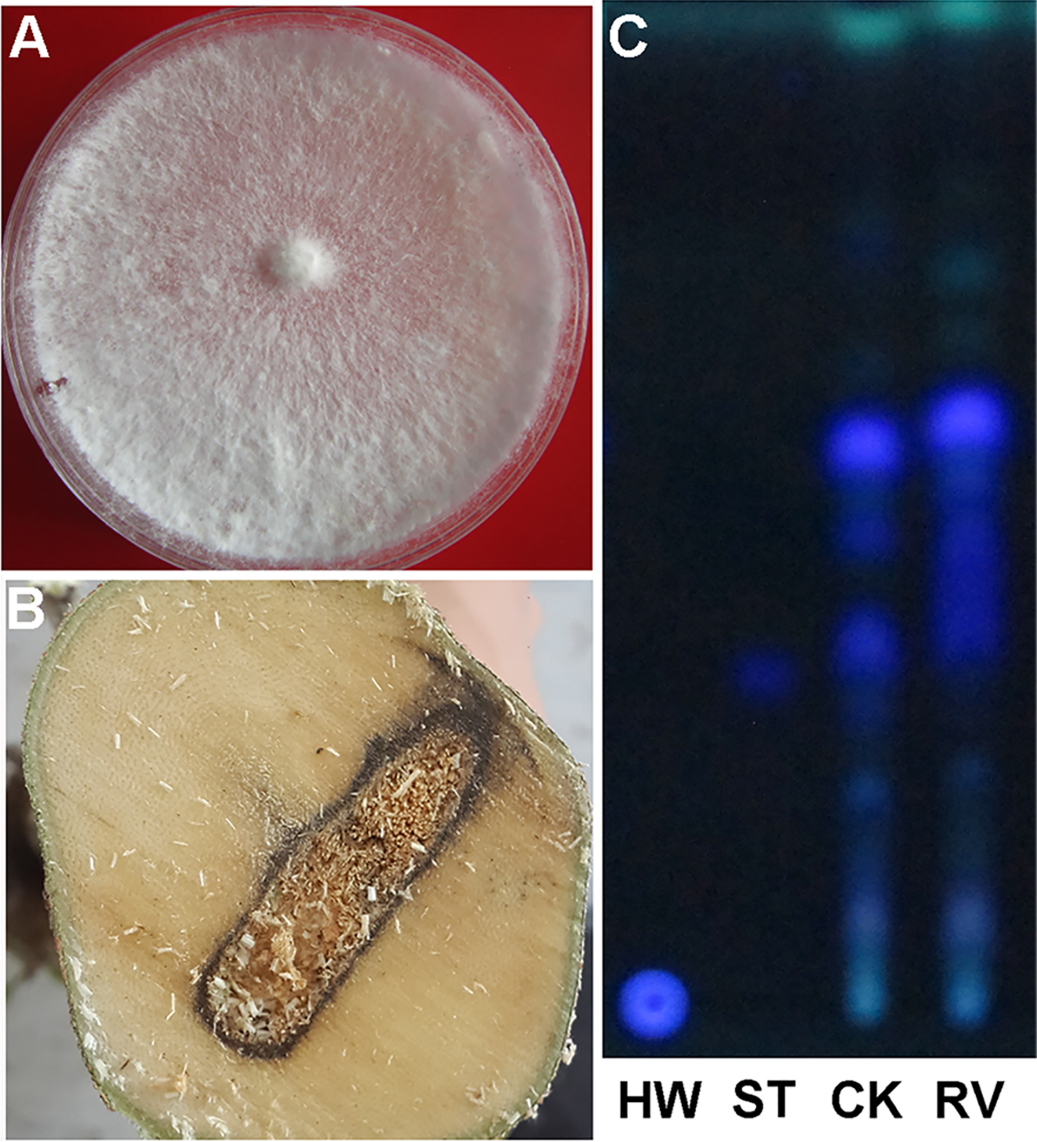
Fungal colonies, the cross sections of the wood injected with fungal fermentation liquid and the TLC plate. A: Colony of *R*. *vinctus*; B: Cross section of the wood injected with *R*. *vinctus* fermentation liquid; C: Spot profile on the TLC plate. HW: Healthy wood; ST: standard 6,7-dimethoxy-2-(2-phenylethyl) chromone; CK: standard agarwood; RV: methanol extracts from the wood injected with *R*. *vinctus* fermentation liquid.

To clarify whether *R*. *vinctus* was capable of promoting agarwood formation, the study selected and cultured one isolate of *R*. *vinctus* (Fig 3A). The fermentation liquid of *R*. *vinctus* was applied in *A*. *sinensis* trees by Agar-Wit technique. Two months later, the trunks were cut down. From the cross-section of the trunks injected with *R*. *vinctus*, it was found that the wood changed to black and obvious agarwood-like dark lines appeared (Fig 3B). The TLC result showed that a bright blue spot developed for the methanol extracts of the wood injected with *R*. *vinctus* fermentation liquid, at the same position as chromone standard 6, 7-dimethoxy-2-(2-phenylethyl) chromone; Additionally, the spot pattern for the methanol extracts of the wood injected with *R*. *vinctus* fermentation liquid was similar to that of the standard agarwood bought from the National Institutes for Food and Drug Control, China (Fig 3C). As chromone is a kind of characteristic compound in agarwood and no chromone is formed in the healthy wood of *A*. *sinensis*, it was primarily deemed that agarwood formation could be induced by *R*. *vinctus*.

### Agar-Sit

In order to gain agarwood formation while avoiding the cutting down of a whole tree and to use fungi for rapid agarwood production, the study developed an efficient method called the Trunk Surface Agarwood-Inducing Technique (Agar-Sit) (S1 Fig). First, a bark of about 50 cm long was uncovered to expose a rectangular surface of the xylem. The bark remained connected with the trunk for later covering (Panel A in S1 Fig). Next, grids (2cm×2cm) of 1.5–2.0 cm deep were made using a knife (Panel B and D in S1 Fig). Several rectangular barks were uncovered from one tree. The distance between an upper and a lower rectangular surface was set to be 20 cm, and that between the opposite ones on different sides of the trunk was 5 cm (Panel C and D in S1 Fig). The fungi liquid was sprayed using a watering pot or smeared using a brush into the grids. The exposed xylem was subsequently covered with the original bark. Six months later, the decay layer on the surface was removed, and the agarwood formed was cut off, leaving the xylem for another run of agarwood induction.

### *R*. *vinctus* promotes agarwood formation by Agar-Sit

**Fig 4:**
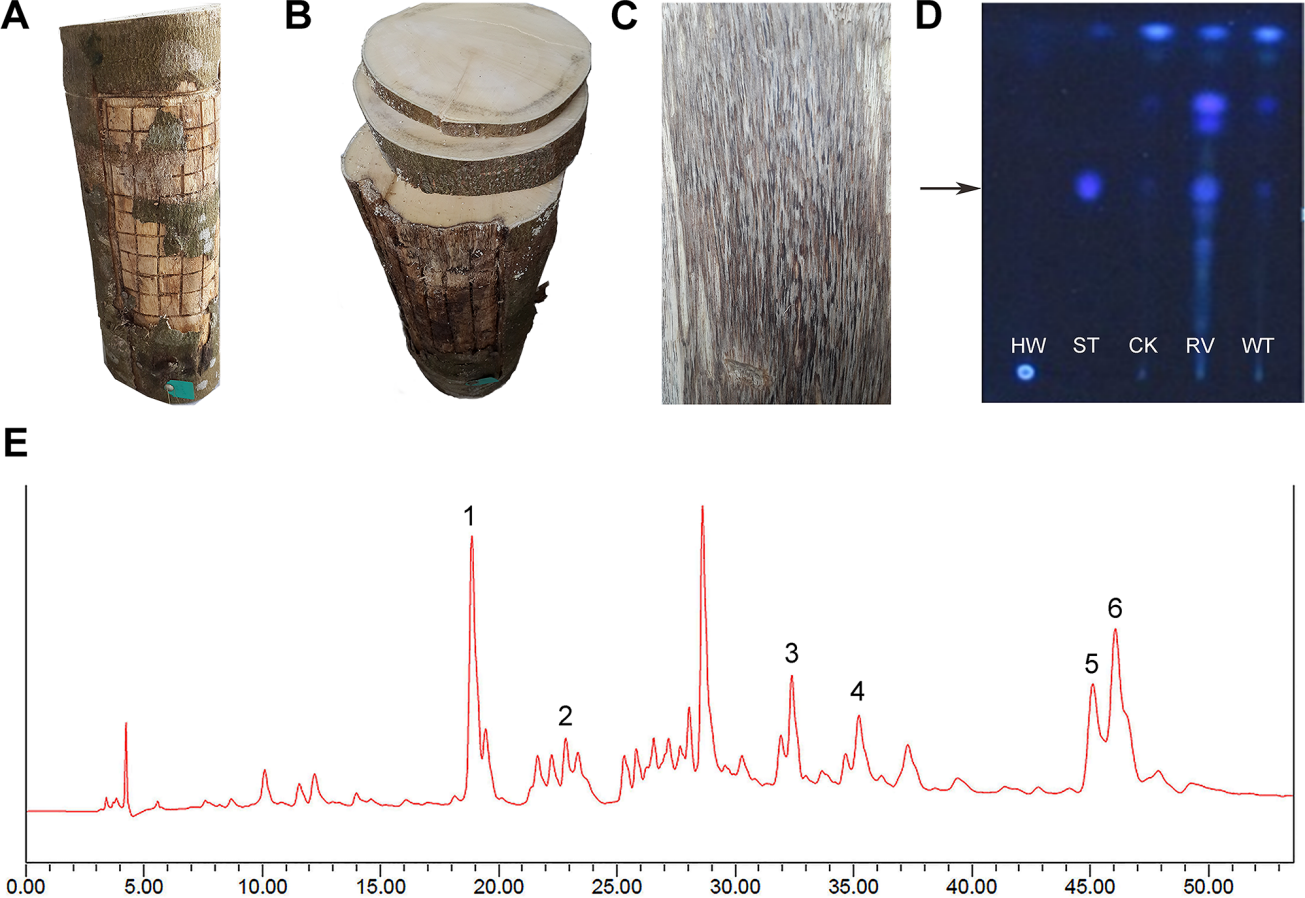
Formation and assay of agarwood with *R*. *vinctus* by Agar-Sit. A: The trunk harvested from the trees 6 months after treatment; B: Thin and black layers of agarwood clearly seen in the cross-sections of the trunk; C: The agarwood layer formed under the decaying surface. D: TLC assay (HW: healthy wood; ST: 6, 7-Dimethoxy-2-(2-phenylethyl) chromone; CK: Wood from the tree without any fungus treatment using Agar-Sit; RV: Agarwood induced by *R*. *vinctus* using Agar-Sit; WT: Wild agarwood). E: Characteristic HPLC chromatogram of chromones from the agarwood induced by *R*. *vinctus* using Agar-Sit. Peak 1: agarotetrol; Peak 3: 8-chloro-2-(2-phenylethyl)-5, 6, 7-tri-hydroxy-5, 6, 7, 8-tetrahydrochromone; Peak 5: 6,4'-dihydroxy-3'-methoxy-2- (2-phenylethyl) chromone.

The trunks treated with *R*. *vinctus* by Agar-Sit method were harvested after six months (Fig 4). Instead of cutting the tree, the formed agarwood was cut off, leaving the tree growing. It can be seen that decaying wood appeared on the surface (Fig 4A). As shown in Fig 4, a thin layer of agarwood can be clearly seen in the cross-section of the trunk (Fig 4B). When removing the decaying layer, the agarwood layer was exposed (Fig 4C). The agarwood is black. The advantages of using Agar-Sit were the induced agarwood was easy for collection, and there was no need to cut down a tree when collecting the agarwood.

### Quality assay of the agarwood by Agar-Sit with *R*. *vinctus*

Chromone is one of the two major characteristic bioactive components of agarwood. No chromone compounds exist in the healthy wood of agarwood-producing trees. In previous research, the identification of existing chromone was considered indicative of agarwood formation. Here the study took 6, 7-Dimethoxy-2-(2-phenylethyl) chromone as a standard (ST, Rf = 0.37), and tested the formed agarwood by the thin layer chromatography (TLC) method. Agarwood induced by *R*. *vinctus* displayed the brightest blue spot in the same position with ST. The control using mere cultural liquid medium instead of *R*. *vinctus* liquid by Agar-Sit method showed a very slight blue spot in the same position with ST, which is a little weaker than a wild agarwood sample (WT) (Fig 4D). The slight spot appeared because the damage to the tree trunk sometimes could result in the slow formation of a little agarwood or agarwood characteristic metabolites. Based on this, it can be assumed that *R*. *vinctus* is capable of promoting agarwood formation.

Generally, high-quality agarwood has high ethanol-soluble extract content. Here the contents of ethanol soluble extracts in the agarwood produced with the *R*. *vinctus* were determined to be about 38.9%, which is far surpassing the requirement (10%) for traditional Chinese medicine listed in Chinese Pharmacopoeia.

According to *Chinese Pharmacopoeia*, agarwood usually has six typical components when extracted with 95% aqueous ethanol and detected by high-performance liquid chromatography (HPLC). The agarwood produced in this study was assayed by HPLC. The results show that the six characteristic peaks corresponding to the six typical components were identical with those in the typical chromatogram presented in *Chinese Pharmacopoeia* (Fig 4E). Three of the six components were clarified, i.e. Agarotetrol (peak 1), 8-chloro-2-(2-phenylethyl)-5, 6, 7-tri-hydroxy-5,6,7,8-tetrahydrochromone (peak 3), and 6,4'-dihydroxy-3'-methoxy-2-(2-phenylethyl) chromone (peak 5). Agarotetrol was quantified to be 0.14% for the RV-treated agarwood, over the required value (0.1%) for traditional Chinese medicine listed in Chinese Pharmacopoeia.

In a GC-MS analysis on essential oil extracted from agarwood produced by Agar-Sit, the main compounds present in agarwood essential oil has been identified as sesquiterpenes. In this work, 15 main sesquiterpene compounds were chosen to compare the quality of agarwood, the relative percentage of total sesquiterpenes was 22.76% for RV samples (Table 2).

**Table 2:** Relative percentage of the components in the essential oil of the agarwood.

No.	Retention Time (min)	Compound	Molecular formula	Area of Percentage
1	15.06	cis-α-Santalol	C_15_H_24_	0.35
2	16.99	[1aR-(1a α,4α,4a β,7bα)]-1a,2,3,4,4a,5,6,7b-Octahydro-1,1,4,7- tetramethyl-1H-Cycloprop[e]azulene	C_15_H_24_	0.37
3	17.15	Octahydro-1,4,9,9-tetramethyl-1H-3a,7-Methanoazulene	C_15_H_26_	1.22
4	17.30	Aromandendrene	C_15_H_24_	0.45
5	17.51	α-Guaiene	C_15_H_24_	0.51
6	17.65	1-methyl-2,4-di(prop-1-en-2-yl)-1-vinylcyclohexane	C_15_H_24_	0.35
7	17.92	[1R-(1-α,3a-β,4-α,7-β)]-1,2,3,3a,4,5,6,7-Octahydro-1,4-dimethyl-7-(1-methylethenyl)-azulene	C_15_H_24_	3.82
8	19.72	4,6,6-Trimethyl-2-(3-methylbuta-1,3-dienyl)-3-oxatricyclo[5.1.0.0(2,4)]octane	C_15_H_24_O	0.66
9	23.13	Caryophyllene-(I1)	C_15_H_24_	0.34
10	23.71	cis-Z-α-Bisabolene epoxide	C_15_H_24_O	0.67
11	25.17	(4aR-cis)-4,4a,5,6,7,8-hexahydro-4a,5-dimethyl-3-(1-methylethylidene)-2(3H)-Naphthalenone	C_15_H_22_	2.26
12	31.79	(-)-Isolongifolol methyl ether	C_15_H_24_	0.63
13	32.24	Longifolene	C_15_H_24_	0.25
14	32.39	(Z,Z)-α-Farnesene	C_15_H_24_	7.01
15	32.81	(+)-(1a-α,3a-β,6a-β,6b-α)-1a,2(1H)-dicarboxaldehyde,3a,4,5,6,6a,6b-hexahydro-5,5,6b-trimethyl-cycloprop[e]indene	C_15_H_20_O	3.87
**Total**				22.76

## Discussion

In the present study, a fungal isolate which invaded deeply into the trunk of *A*. *sinensis* was first reported and was capable of promoting agarwood formation. The fungus was applied to the originally invented Agar-Sit method which induced high-quality agarwood. The discovery of the isolate of *R*. *vinctus* was due to the observation that some wild fungi could enter the inner of wood and induce agarwood formation. So the study isolated the fungi from different layers from a special sample with microbial infection characteristics. The purpose of this study was to find suspicious fungi from different layers and confirm suspicious fungi can promote agarwood formation. Fortunately, *R*. *vinctus* were isolated and confirmed that it possesses strong infection ability to induce agarwood formation. *R*. *vinctus* is a kind of wood-degrading microorganism belonging to white rot fungus, which often causes the death of crops [23]. As already reported, white rot fungus degrades cellulose and lignin of plant cells mainly through its highly efficient enzymatic system, including the activities of lignin peroxidase (LiP), manganese peroxidase (MnP) and laccase [24]. As agarwood formation is considered as a result of defense response when trees confront wounding, it may be assumed that *R*. *vinctus* wounds trees by biodegrading lignin, thus inducing defense responses of these trees. This agrees with the hypothesis that agarwood formation results from a tree’s defense response to diverse damages, including mechanical wound, microbial invasion, and chemical stresses [25].

In order to induce agarwood formation, a quite number of agarwood-inducing fungi had been reported and many methods for the fungi had been used [7, 13, 26]. Maybe because of the weak infectivity of those fungi, it was very difficult to penetrate thick wood to induce agarwood formation. When such fungi alike were inoculated into the holes drilled in the trunk of trees, the agarwood could only form in a very thin layer near the surface of holes. In addition, much wood was not efficiently used for agarwood formation when the method of drilling holes was used. Therefore, the prominent problem in innovating agarwood production is to find high-infectivity agarwood-promoting fungus and to explore corresponding high-efficiency fungi application method. Here, it was reported that the successful isolation of *R*. *vinctus* spread not only in the agarwood layer but also in the healthy white wood layer beneath the agarwood layer. Therefore inducing agarwood formation when it was applied, it displayed high infectivity and could invade past the deep wood of the trunk. According to the characteristics of the isolate of fungus the research obtained, the novel fungus application method, Agar-Sit was invented. It could be used with *R*. *vinctus*, as well as any other agarwood-promoting fungi. However, with respect to the available information, Agar-Sit is the best method for applying the fungi in agarwood induction. The major advantages of Agar-Sit are reflected in its promoting agarwood formation in the trunk surface so that it is very easy to collect agarwood also with the non-obligation to destroy the whole tree. Comparatively, high-quality agarwood could be efficiently obtained by using Agar-Sit and the high infectious isolate of *R*. *vinctus*. In addition, Agar-Sit could be combined and used with Agar-Wit, the whole-tree agarwood-inducing technique, which the laboratory developed. It is now widely used in agarwood production. When the two methods were combined, agarwood formed both inside and on the surface of the trunk, increasing the yield.

Previous reports on agarwood production using fungus usually adopted digging holes method to load fungi cultures, fungi fermentation liquid or fungi powder. Due to the prominent invasive capability of the isolate of *R*. *vinctus*, the study considered whether it could be used directly from the trunk surface of the wood because Agar-wit cannot use the trunk surface. Based on this the Agar-sit method was designed. The vital issue was regarding the method to maintain the fungus staying on the surface of the wood for certain time so as to ensure the fungus had enough time for propagation and invasion. First, a wound is placed on the surface of the trunk to facilitate fungal attachment. Then the medium is sprayed on the trunk which helps the growth of the *R*. *vinctus*. Finally, fungi is sprayed, fungi fermentation liquid or fungi powder.

The Agar-wit method that was invented earlier is being successfully and widely used presently. But a problem still exists, large parts of healthy woods are not efficiently used (S2 Fig). The yield of agarwood can further be elevated, by combining Agar-Sit method with *R*. *vinctus* and Agar-Wit method. This way the wood can be used fully and the yield of agarwood can be elevated significantly. Although Agar-Sit can promote high-quality agarwood formation, the method of Agar-Sit with *R*. *vinctus* still can be improved, such as the shortening of setting time of the wound surface on the trunk, so that the fungi are easy to carry and so on.

## Supporting information

S1 Fig Illustration of the Agar-Sit method. Panel A: A bark was uncovered; Panel B: the vertical lines of grids (2 cm spacing) were made; Panel C: more than one piece of bark were uncovered in a single tree; Panel D: the horizontal lines of grids (2cm×2cm) were made with a knife.
(TIF)

S2 Fig The whole-tree agarwood-inducing technique (Agar-Wit). Panel A: Agarwood induced by agarwood inducer; Panel B: Health wood.
(TIF)

## References

[1] Ito M, Honda G. Taxonomical identification of agarwood-producing species. Nat Med. 2005; 59: 104–112.

[2] Persoon GA, Van Beek HH. Growing ‘the wood of the gods’: agarwood production in southeast Asia In: Snelder DJ, Lasco RD, editors. Smallholder tree growing for rural development and environmental services: lessons from Asia. Dordrecht: Springer Netherlands; 2008 pp. 245–262.

[3] Yagura T, Shibayama N, Ito M, Kiuchi F, Honda G. Three novel diepoxy tetrahydrochromones from agarwood artificially produced by intentional wounding. Tetrahedron Lett. 2005; 46: 4395–4398.

[4] Gao ZH, Wei JH, Yang Y, Zhang Z, Xiong HY, Zhao WT. Identification of conserved and novel microRNAs in *Aquilaria sinensis* based on small RNA sequencing and transcriptome sequence data. Gene. 2012; 505: 167–175. doi: 10.1016/j.gene.2012.03.072 22521867 10.1016/j.gene.2012.03.072

[5] CITES. Proposals fro amendment of Appendices I and II (CoP13 Prop.49). In: Proceedings of Thirteenth Meeting of the Conference of the Parties [Internet]. Bangkok (Thailand): 2–14 October 2004. Available from: https://www.cites.org/eng/cop/13/prop/index.php

[6] Naef R. The volatile and semi-volatile constituents of agarwood, the infected heartwood of *Aquilaria* species: a review. Flavour Frag J. 2015; 26: 73–87.

[7] Chen XY, Sui C, Liu YY, Yang Y, Liu PW, Zhang Z, et al Agarwood formation induced by fermentation liquid of *Lasiodiplodia theobromae*, the dominating fungus in wounded wood of *Aquilaria sinensis*. Curr Microbiol. 2017; 74: 460–468. doi: 10.1007/s00284-016-1193-7 28224223 10.1007/s00284-016-1193-7

[8] Wei JH, Yang Y, Zhang Z, Meng H, Feng JD, Gan BC. Production of agarwood in Aquilaria sinensis trees via transfusion technique. China patent CN101755629 B. 2010.

[9] Burkill IH. A dictionary of the economic products of the Malay Bhattacharya on the formation and development of agar in *Aquilaria agalocha*. Sci Cul. 1935: 240–242.

[10] Bhattacharya B, Dutta A, Baruah HK. On the formation and development of agarol in *Aquilaria agalocha*. Sci Cult. 1952; 18: 240–241.

[11] Guangdong Institute of Botany. Preliminary disclosure secret of agarwood formation of *Aquilaria* sp. Acta Botanica Sinica. 1976; 18: 287–290. in Chinese.

[12] Qi SY, Lin LD, Ye QF. Benzylacetone in agarwood and its biotransformation by *melanotus flavolivens*. Chin J Biotech. 1998; 14: 464–466. in Chinese.

[13] Subehan, Ueda J, Fujino H, Attamimi F, Kadota S. A field survey of agarwood in Indonesia. J Tradit Med. 2005; 22: 244–251.

[14] Xu WN. Evaluation on key technology of fungi infection induced aloes-forming effect and preliminary research on the mechanism of the eaglewood formation. M.Sc. Thesis, Guangdong Pharmaceutical University. 2011. in Chinese.

[15] Chen XY, Liu YY, Liu PW, Peng DQ, Wei JH. Study on biological characteristics of two strains of *Lasiodiplodia theobromae* promoting agarwood formation. Acta Agriculturae Jiangxi. 2017; 29: 95–98. in Chinese.

[16] Huang H. An agarwood promoting reagent and its usage in Aquilaria sinensis. China patent CN102499264 A. 2013.

[17] Guo SX, Cui JL, Chen XM, Wang CL, Meng ZX. Three fungi capable of inducing Aquilaria sinensis to produce agilawood. China patent CN102649939 A. 2013.

[18] Blanchette RA, Van Beek HH. Cultivated agarwood. USA patent US7638145 B2. 2009.

[19] Chen XY, Qi YD, Wei JH, Zhang Z, Wang DL, Feng JD, et al Molecular identification of endophytic fungi from medicinal plant *Huperzia serrata* based on rDNA ITS analysis. World J Microbiol Biotechno. 2011; 27: 495–503.

[20] Liu YY, Chen HQ, Yang Y, Zhang Z, Wei JH, Meng H, et al Whole-tree agarwood-inducing technique: an efficient novel technique for producing high-quality agarwood in cultivated *Aquilaria sinensis* trees. Molecules. 2013; 18: 3086–3106. doi: 10.3390/molecules18033086 23470337 10.3390/molecules18033086 PMC6270329

[21] Zhang Q, Huo HX, Gu YF, Zhao YF, Li J, Tu PF. HPLC-DAD characteristic chromatogram of Chinese eaglewood. Chinese Pharmaceutical Journal. 2015; 50: 213–216. in Chinese.

[22] Commission of Chinese Pharmacopoeia. Chinese Pharmacopoeia. Beijing (China): Chinese Medicine Science and Technology Publishing House Press; 2010:185–186

[23] Norhaslida R, Halis R, Lakarim L, Danial MI, Low JC, Naimah MS. Chemical alteration of banana pseudostems by white rot fungi. Biomass and Bioenergy. 2014; 61: 206–210.

[24] Mohamed R, Lim MT, Halis R. Biodegrading ability and enzymatic activities of some white rot fungi on kenaf *(Hibiscus cannabinus*). Sains Malaysiana. 2013; 42: 1365–1370.

[25] Zheng Z, Yun Y, Wei JH, Hui M, Sui C, Chen HQ. Advances in studies on mechanism of agarwood formation in *Aquilaria sinensis* and its hypothesis of agarwood formation induced by defense response. Chin Tradit Herbal Drugs. 2010; 41: 156–159. in Chinese.

[26] Faizal A, Esyanti RR, Aulianisa EN, Iriawati, Santoso E, Turjaman M. Formation of agarwood from *Aquilaria malaccensis* in response to inoculation of local strains of *Fusarium solani*. Trees. 2017; 31: 189–197.

