# Supplementary figures and images for: Trunk surface agarwood-inducing technique with Rigidoporus vinctus: An efficient novel method for agarwood production
Source: PLoS One. 2018 Jun 1;13(6):e0198111. doi: 10.1371/journal.pone.0198111 (PMC5983524; doi:10.1371/journal.pone.0198111)

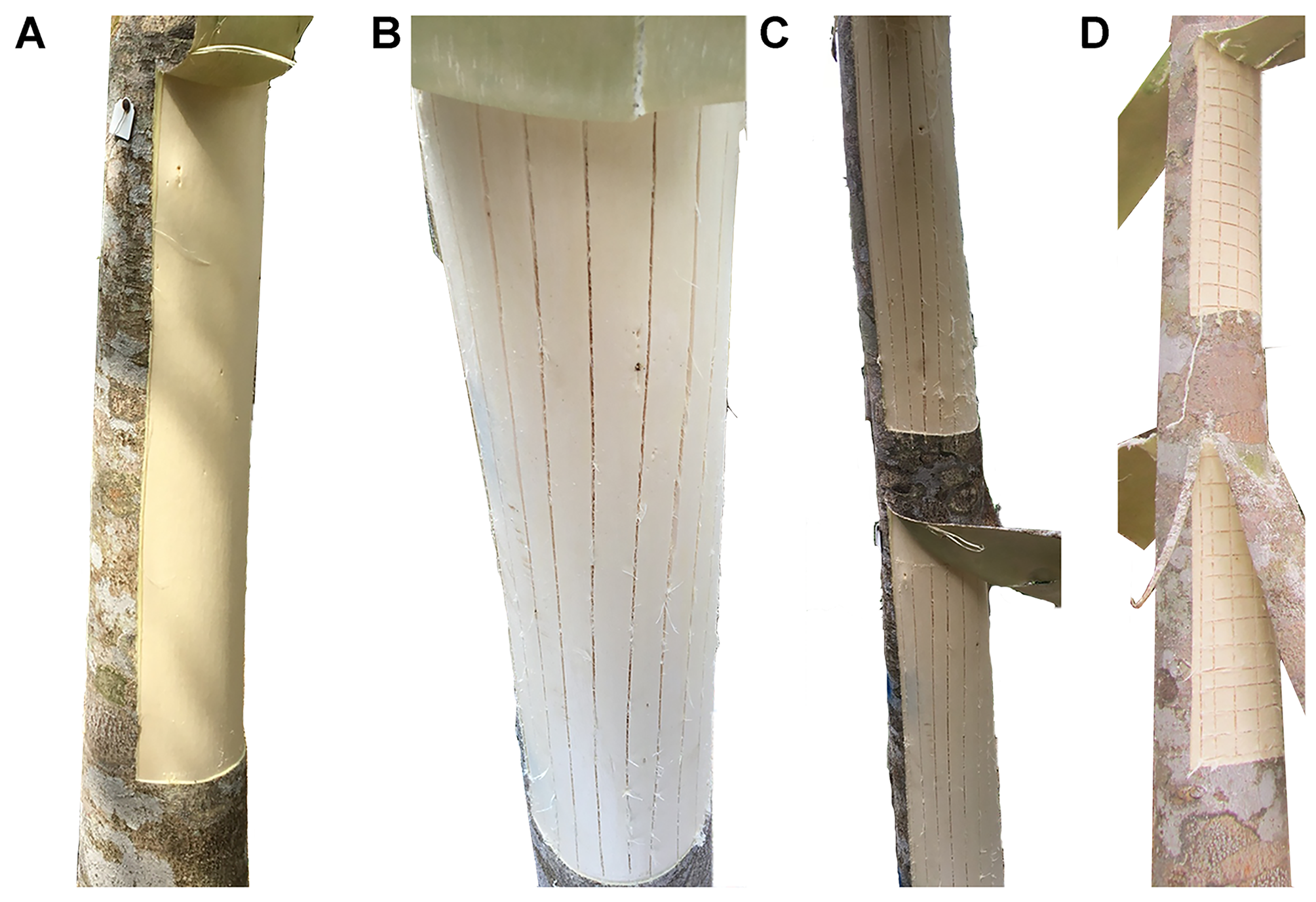

Supplement: S1 Fig — Panel A: A bark was uncovered; Panel B: the vertical lines of grids (2 cm spacing) were made; Panel C: more than one piece of bark were uncovered in a single tree; Panel D: the horizontal lines of grids (2cm×2cm) were made with a knife. (TIF) [file pone.0198111.s001.tif]

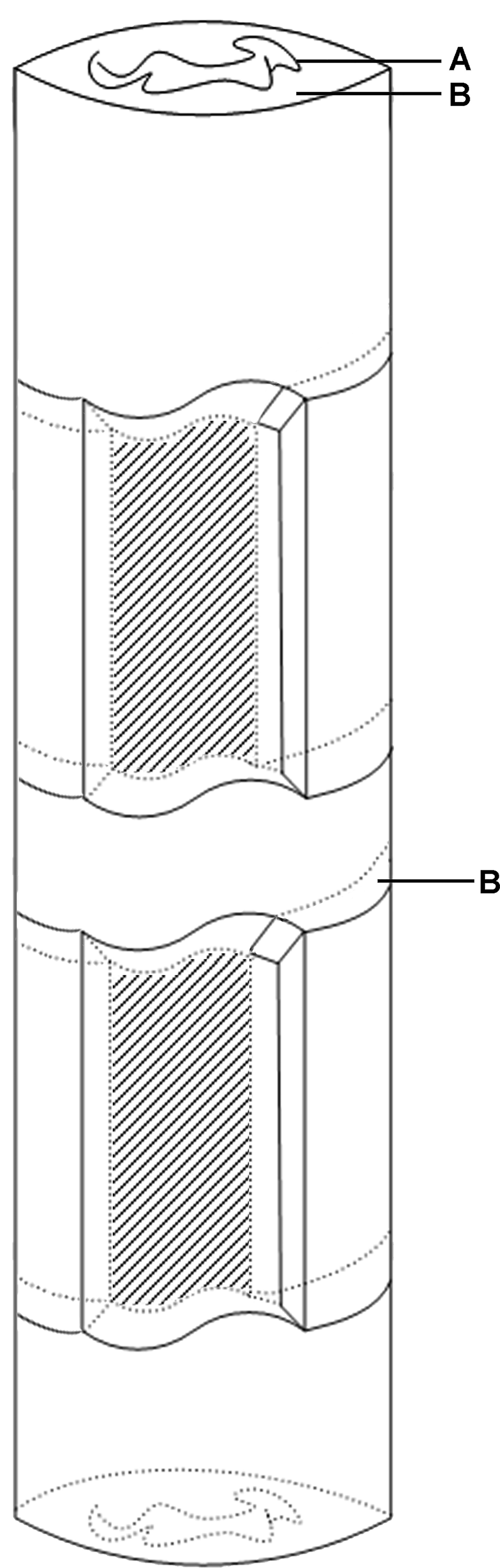

Supplement: S2 Fig — Panel A: Agarwood induced by agarwood inducer; Panel B: Health wood. (TIF) [file pone.0198111.s002.tif]
